# The faster you decide, the more accurate localization is possible: Position representation of “curveball illusion” in perception and eye movements

**DOI:** 10.1371/journal.pone.0201610

**Published:** 2018-08-06

**Authors:** Hiroshi Ueda, Naotoshi Abekawa, Hiroaki Gomi

**Affiliations:** NTT Communication Science Laboratories, Nippon Telegraph and Telephone Co., Kanagawa, Japan; UCLA, UNITED STATES

## Abstract

When the inside texture of a moving object moves, the perceived motion of the object is often distorted toward the direction of the texture’s motion (motion-induced position shift), and such perceptual distortion accumulates while the object is watched, causing what is known as the curveball illusion. In a recent study, however, the accumulation of the position error was not observed in saccadic eye movements. Here, we examined whether the position of the illusory object is represented independently in the perceptual and saccadic systems. In the experiments, the stimulus of the curveball illusion was adopted to examine the temporal change in the position representation for saccadic eye movements and for perception by varying the elapsed time from the input of visual information to saccade onset and perceptual judgment, respectively. The results showed that the temporal accumulation of the motion-induced position shift is observed not only in perception but also in saccadic eye movements. In the saccade tasks, the landing positions of saccades gradually shifted to the illusory perceived position as the elapsed time from the target offset to the saccade “go” signal increased. Furthermore, in the perception task, shortening the time between the target offset and the perceptual judgment reduced the size of the illusion effect. Therefore, these results argue against the idea of dissociation between saccadic and perceptual localization of a moving object suggested in the previous study, in which saccades were measured in a rushed way while perceptual responses were measured without time constraint. Instead, the similar temporal trends of these effects imply a common or similar target representation for perception and eye movements which dynamically changes over the course of evidence accumulation.

## Introduction

It is natural to think that our visually guided actions are produced based on what we perceive from the environment. However, a great deal of research has shown that even with the same visual input, stimulus-response characteristics given by consciously perceived information do not necessarily match those observed in visually guided motor actions (e.g., [1,2]). These results have been interpreted as evidence of the so-called two-streams hypothesis that there are two distinct kinds of vision, namely "vision for perception" and "vision for action" [3–8], but this has been a subject of considerable debate and no consensus has yet been reached [9–13].

One of the ongoing questions regarding the perception-action coupling/dissociation is whether both systems share the same neural positional representation [14–18]. To investigate this question, a perceptual illusion called *motion-induced position shift* (MIPS) has often been used. MIPS is a phenomenon in which the perceived position of a stationary object with internal (e.g., texture) motion is shifted in the direction of motion [19–21]. Previous studies have shown that the effect is more pronounced at the visual periphery than at the fovea [20] and is dependent on the spatial and temporal frequency of the internal motion [22]. The advantage of using MIPS stimulation in perception-action studies is that visuomotor localization of this type of stimulus allows us to examine whether visually guided movements are generated based on the illusory perceived or actual physical position information. Indeed, recent studies have shown that local motion signals bias both perception and actions (manual pointing and saccadic eye movements) in a similar fashion, suggesting that they rely on shared target position representation ([23,24], but see [14]).

In contrast to the conventional MIPS (or *single-drift stimuli*: a stationary object with internal pattern motion), however, a recent study by Lisi and Cavanagh [25] has reported that *double-drift stimuli* (a moving object with internal pattern motion) have a dramatically different effect on perception and eye movements. A double-drift stimulus, which is also known as the curveball illusion, strikingly alters the perceived direction of a moving Gabor from its actual trajectory towards the direction of internal pattern motion [25–27]. This stimulus preserves the basic characteristics of single-drift stimuli, such as greater effect at the visual periphery than the fovea [27,28] or with higher temporal frequency than lower frequency [27]. However, it also causes the accumulation of MIPS over observing time, which results in a dramatic distortion of the perceived position [25,27,28]. Interestingly, Lisi and Cavanagh [25] have reported that while internal motion slightly shifted saccade landings at each point in time, the large distortion observed in perceptual localization was not observed in saccadic localization.

Lisi and Cavanagh [25] demonstrated clear perception–action dissociation in target representation for double-drift stimuli. However, assuming that perceptual distortion in double-drift stimuli is caused by a temporal accumulation of MIPS, a fundamental question will arise as to whether the perception-action dissociation is independent of or dependent on the duration of the accumulation process. In the previous studies on double-drift stimuli [25–27], perceptual responses were measured without temporal constraint (i.e., observers answered whenever they decided to), while saccades were measured in an "as soon as possible" manner. This difference in response manner could be allowed based on a naive assumption that these responses do not change afterward. However, this comparison of target representation at different times in visual processing (e.g., offline perception versus online saccades) can lead to differences in target representation for perception and action. Indeed, previous studies on MIPS have shown that the magnitude of the illusion effect depends critically on when the response is made regardless of response modality [14,23,24]. Therefore, in order to rigorously compare the target representation for perception and action, it would be necessary to characterize temporal changes in the effect of double-drift stimuli on perception and saccade.

To address this issue, the present study examined the temporal changes in the target representation of double-drift stimuli for saccadic eye movements (Experiment 1) and for perception (Experiment 2). In both experiments, we presented a double-drift stimulus that descended for 2 s and disappeared. In Experiment 1, we asked participants to make saccades towards the final target position when a go signal was presented while changing the temporal gap from the target disappearance to the saccade go signal. The results showed that the temporal gap significantly affects saccade landing points—the longer the temporal gap, the larger the deviation in the direction of internal motion. This suggests that the saccade system does not always access physical object’s location, but rather that, after a sufficient accumulation process, it also relies on the position representation integrated with motion signals as in perception. Interestingly, in Experiment 2, as in the case of saccades, the size of the perceptual illusion increased with the prolongation of the temporal gap (between the target disappearance and a visual reference presentation). These results suggest that the saccadic and perceptual systems rely on shared or similar neural representations of the target location, which dynamically changes in the integration process with the internal representation of position history. Some of these data have been presented in abstract and poster form [29].

## Experiment 1

Experiment 1 consisted of two tasks for perception and saccade. In the perception task, to assess whether the illusion actually occurs on position (rather than motion direction) perception even under conditions with a time constraint, the perceived final position of a double-drift stimulus at a certain point in time after the stimulus offset was objectively determined by a two-alternative forced choice paradigm (see Methods section for details). In the saccade task, in order to investigate whether the time delay before saccades has any influence on the saccadic localization, the saccade landing positions were measured while varying the time elapsed from the target offset to the saccade initiation. If distinct neural representations of target position were used in the perceptual and saccadic systems as in the previous study [25], it is expected that the accumulation effect of MIPS is observed only in perception but not in saccadic eye movements even when the time gap is increased.

### Methods

#### Participants

Sixteen paid volunteers (12 women and 4 men, aged 22–44 years; *M* = 35.6, *SD* = 7.73) with normal or corrected-to-normal vision participated in both the perception and saccade tasks. All gave written informed consent prior to each experiment. All methods employed in this study were approved by the Ethics and Safety Committees of NTT Communication Science Laboratories and were in accordance with the Declaration of Helsinki. The protocol number of the Ethics and Safety Committees of NTT Communication Science Laboratories is H27-016.

#### Experimental setting and apparatus

The experiment was conducted in a dark room. Visual stimuli were generated by MATLAB (MathWorks) using the Psychophysics Toolbox extensions [30,31] and were presented on a gamma-corrected 22-inch CRT monitor with a resolution of 1024 × 768 pixels and a refresh rate of 120 Hz. A chin rest was used to stabilize participants’ head and eye level at the center of the screen with a viewing distance of 44 cm. Eye movements were recorded monocularly with a tower-mounted EyeLink 1000 (SR Research) at a sampling rate of 1 kHz using the EyeLink Toolbox extensions [32] of MATLAB. The eye tracker was calibrated using nine reference points before each block of trials.

#### Stimuli and procedure

**Fig 1:**
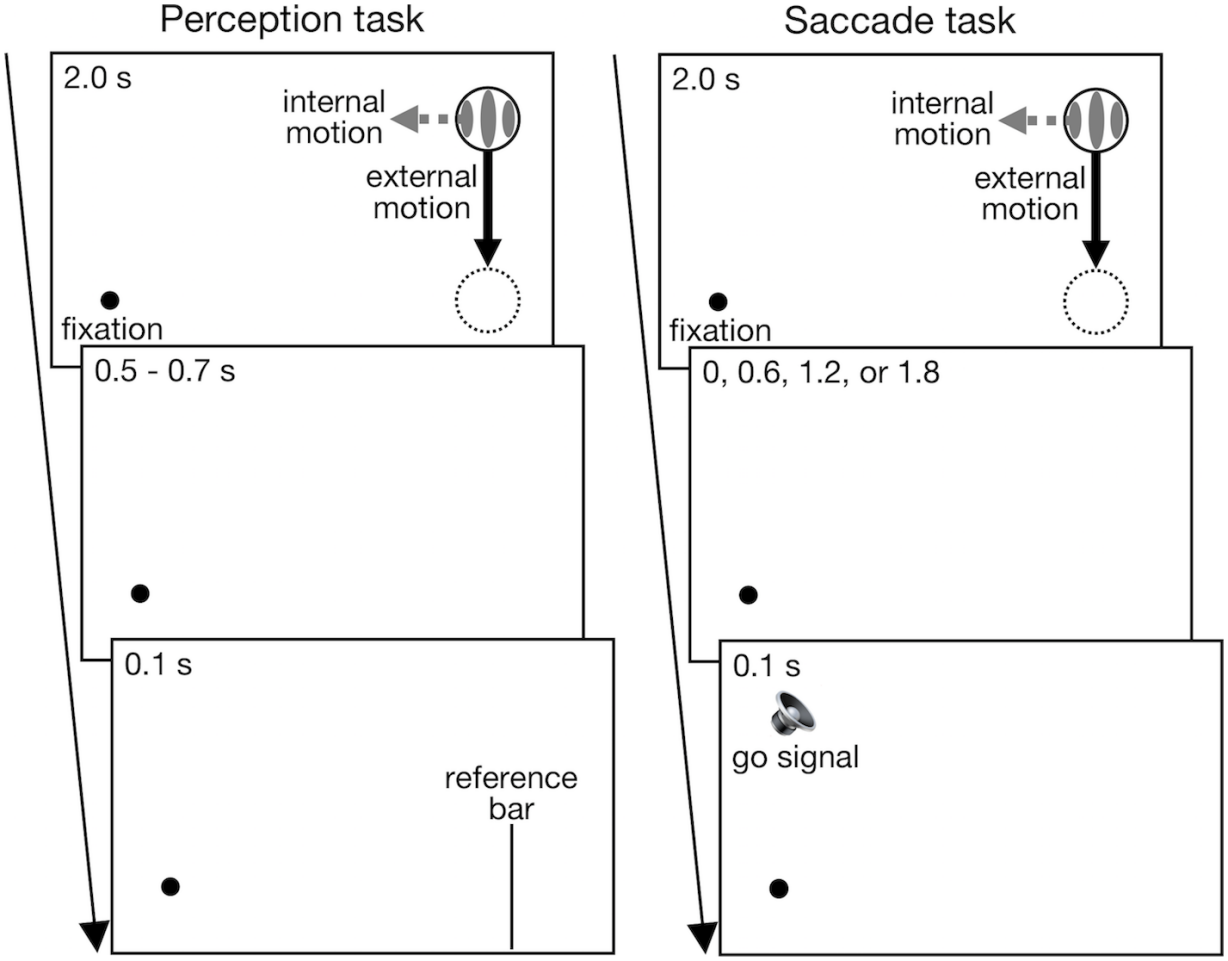
Schematic representations of the perception and saccade task used in Experiment 1. In both tasks, participants started a trial by pressing the spacebar while keeping their gaze on the fixation dot. The Gabor target moved vertically (external motion: 2°/s; 2.0 s) with or without the grating drift (internal motion: leftward or rightward; 3 Hz) and disappeared at the fixation level. In the perception task, after a random time interval of 0.5–0.7 s, a vertical reference bar (10°; 0.1 s) appeared at one of the seven horizontal locations (-3°, -2°, -1°, 0°, +1°, +2°, or +3° relative to the target position), and participants made a 2-AFC indicating whether the reference bar was located to the left or right of the final target position. In the saccade task, after a delay of 0, 0.6, 1.2, or, 1.8 s, an auditory “go” signal (1000 Hz; 0.1 s) was presented, and participants made a saccade to the final target position.

Experiment 1 consisted of perception and saccade tasks as shown in Fig 1. The task order was counterbalanced among participants. In both tasks, stimuli were presented on a uniform gray background (22.6 cd/m^2^). The target Gabor patch was created by multiplying a vertical sinusoidal grating (2 cycles/°, 100% Michelson contrast) by a two-dimensional Gaussian contrast envelope with a standard deviation of 0.1°.

In both tasks, each trial started when the participants pressed the spacebar while keeping their gaze on a central fixation point (a black dot: 0.2° in diameter). The Gabor target then appeared 4° above the fixation with one of five horizontal eccentricities (8°, 9°, 10°, 11°, or 12°) to the right of the fixation and started to move vertically with a speed of 2°/s (external motion) until it disappeared at the fixation level (e.g., S1 Movie). The grating of the Gabor target either drifted with a temporal frequency of 3 Hz in a horizontal direction (leftward and rightward internal motion conditions) or remained stationary (control condition).

In the perception task, at a random time interval of 0.5–0.7 s after the target offset, a vertical reference bar (a black line: 10°) appeared for 100 ms at one of seven horizontal locations: -3°, -2°, -1°, 0°, +1°, +2°, or +3° relative to the final target position. The participants were asked to make a two-alternative forced choice indicating whether the reference bar was located to the left or right of the final target position by pressing the left or right arrow key, respectively. Each participant performed 420 trials, divided into four blocks. Each block consisted of all possible combinations of the three internal motion conditions, five external motion paths, and seven reference bar locations (105 trials). The order of stimulus presentation was randomized across trials. Participants performed several practice trials before the test trials to familiarize themselves with the task. During test trials, no feedback about correct or incorrect responses was provided. The trial was immediately aborted when the gaze shifted away from the fixation point (>1.5°) or an eye blink occurred before the disappearance of the reference bar. Drift correction of the eye tracker was also performed after every three aborted trials. Aborted trials were repeated at a random time later within the block.

In the saccade task, an auditory “go” signal (1000 Hz; 0.1 s) was presented with a time delay of 0, 0.6, 1.2, or 1.8 s from the target disappearance. The participants were asked to make a saccade to the final target location as quickly as possible after the “go” signal. Each participant performed 420 trials, divided into seven blocks. Each block consisted of all possible combinations of the four time delays, three internal motion conditions, and five external motion paths (60 trials). The order of stimulus presentation was randomized across trials. Participants performed 30 practice trials before the test trials. The trial was immediately aborted and repeated at a random time later within the block if the participants blinked before giving a response (defined as when the participants shifted gaze more than 1.5° away from the fixation point). Those trials with a response assumed to be an anticipatory response (< 100 ms after the go signal) or a lack of alertness (> 600 ms after the go signal) were also aborted online and repeated at a random time later within the block. Drift correction was again performed after every three aborted trials.

#### Data analysis

In the perception task, for each participant and internal motion condition, the mean proportion of responses that ‘the bar appeared on the right’ was plotted as a function of the horizontal position of the reference bar relative to the final target position and fitted with a logistic function. The reference bar position that would yield the equal probability of “right” and “left” responses (i.e., the point of subjective equality (PSE), corresponding to the 0.5 level of the logistic psychometric function) was regarded as the perceived horizontal position of the final target position. To estimate the size of the perceptual illusion for each participant, we first computed the difference in the PSE of between the rightward and control condition (rightward internal motion effect) and between the leftward and control condition (leftward internal motion effect). The average of the absolute value of the rightward and leftward internal motion effect was used as the size of the illusion for statistical analyses. The data for one participant who did not show a reliable effect of the illusion in the perception task (i.e., obtained a negative effect size of the illusion) were excluded from the analysis of Experiment 1.

In the saccade task, the initiation of saccades was defined as the first time the eye movement exceeded a velocity threshold of 22°/s and an acceleration threshold of 3800°/s^2^ after the “go” signal. Trials were discarded from the analysis if the saccade end position (horizontal or vertical) relative to the final target position had deviated more than 1.5 times the interquartile range from the 25th or 75th percentile of the data for each internal motion and delay condition (boxplot outlier identification procedures). With this criterion, less than 4% of the trials were excluded. To estimate the size of the “saccadic illusion”, we first computed the mean horizontal saccade landing position for each internal motion and delay condition, and then took the difference in the mean landing positions of between the rightward and control condition (rightward internal motion effect) and between the leftward and control condition (leftward internal motion effect). As in the case of the perceptual illusion, the size of the saccadic illusion was defined as the average of the absolute value of the rightward and leftward effects.

### Results and discussion

**Fig 2:**
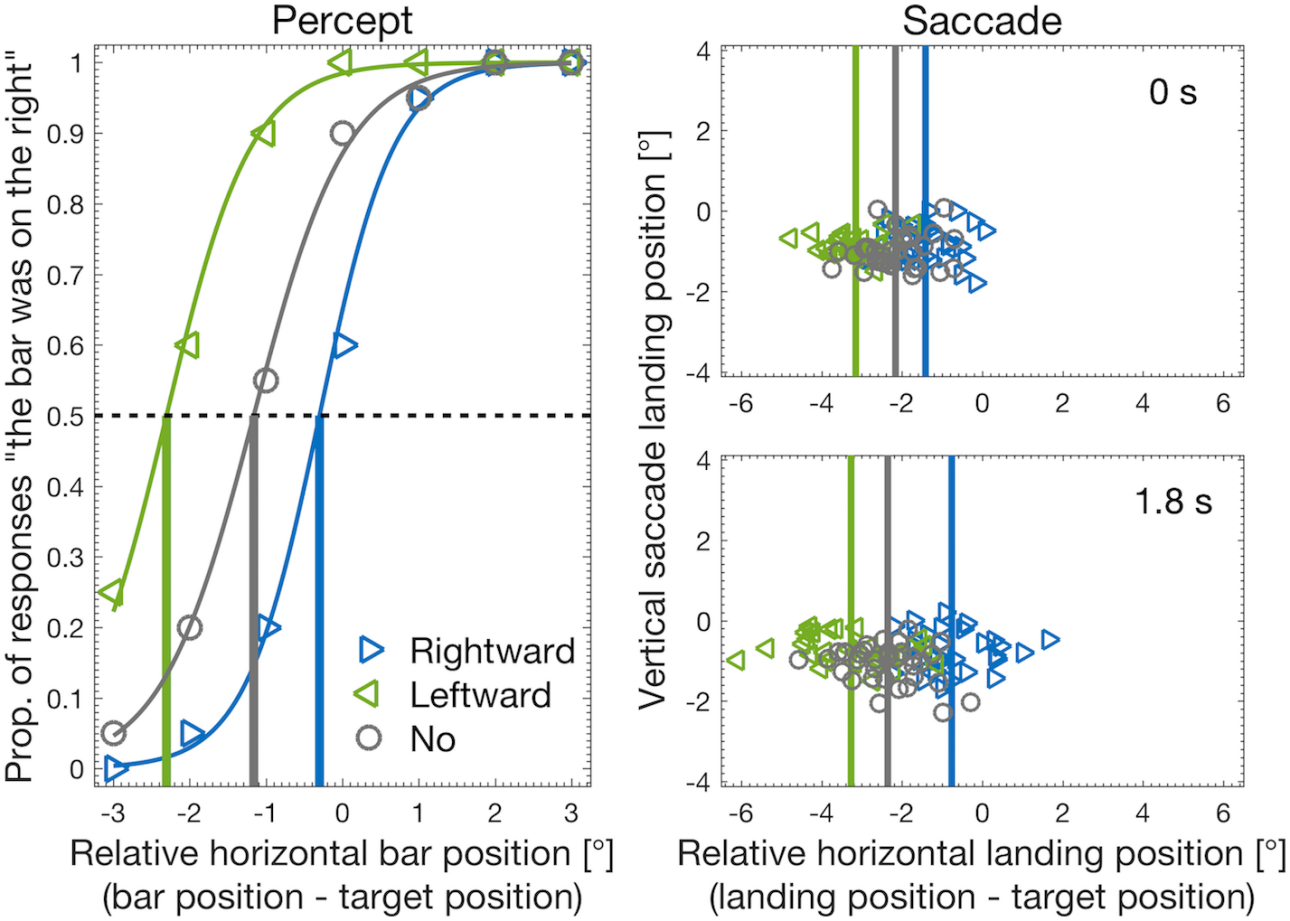
Example of results of Experiment 1 obtained from a single participant. Perception task: psychometric curves for the proportion of the responses that judged the reference bar appeared on the right side of the final target position as a function of the relative reference bar position to the final target position. Vertical lines indicate the PSE of each internal motion condition. Saccade task: saccade landing positions (relative to the final target positions) for two delay conditions (0 and 1.8s). Vertical lines indicate the mean horizontal landing positions of each internal motion condition.

An example of the results for the perception and saccade tasks from a single participant are shown in Fig 2, where the measured perceived position (i.e., PSEs of the psychometric curves) and saccade landing positions for each internal motion condition (rightward, leftward, no motion) are indicated by the vertical blue, green, and gray lines, respectively. The perceived position and saccades were clearly influenced by the internal grating motion of the Gabor target: the perceived horizontal target position and the mean horizontal saccade landing position were biased toward the direction of internal motion.

**Fig 3:**
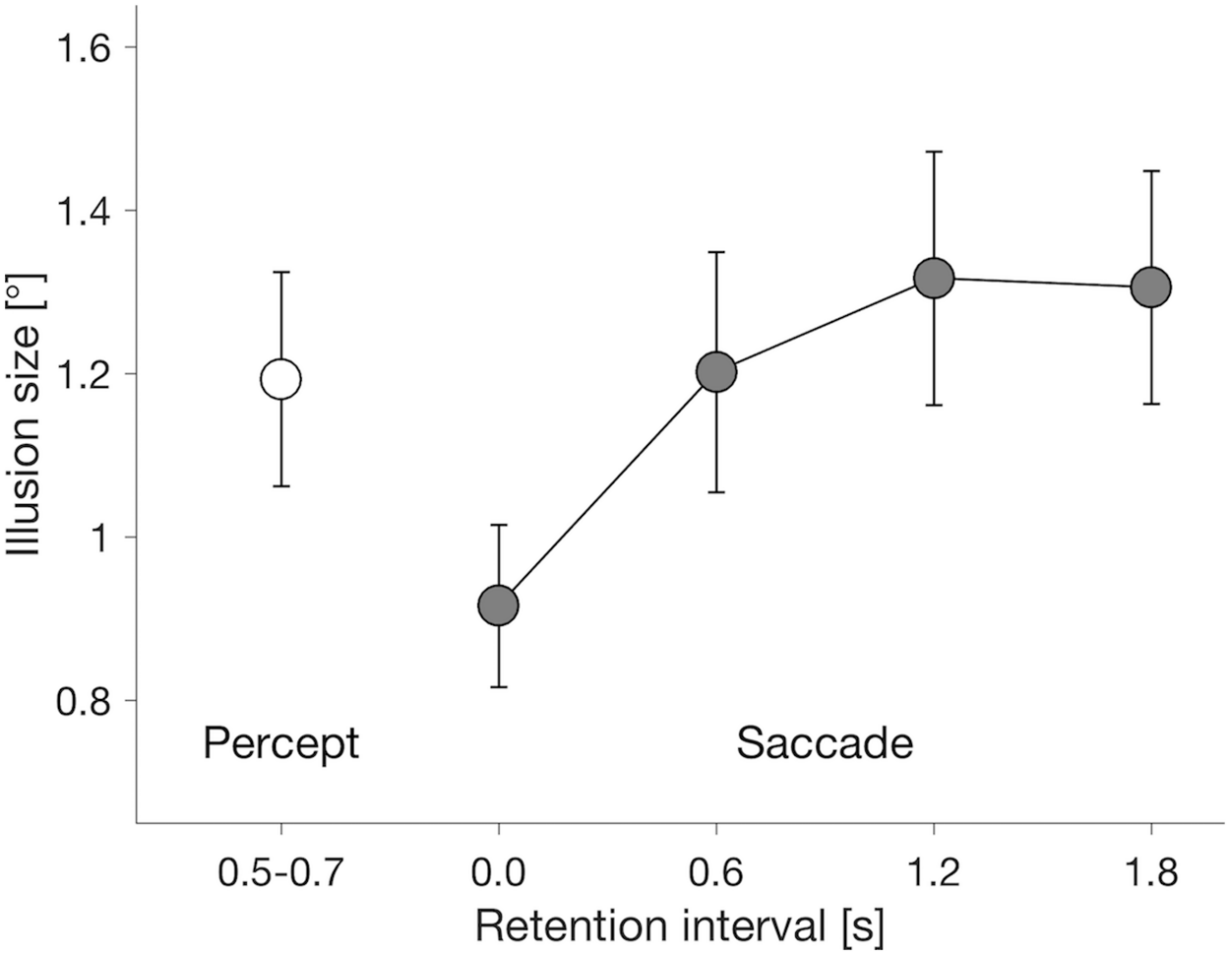
Mean illusion sizes for perception (open circle) and saccade with four delay conditions (filled circles: 0, 0.6, 1.2, and 1.8 s). Error bars indicate standard errors of the means (*N* = 15).

Fig 3 shows the means of the effect of the internal motion (i.e., the size of the illusion) for the perception tasks (the left-most point: *M* = 1.19°, *SD* = 0.51°) and saccade tasks with a delay of 0 s (*M* = 0.92°, *SD* = 0.38°), 0.6 s (*M* = 1.20°, *SD* = 0.57°), 1.2 s (*M* = 1.32°, *SD* = 0.60°) and 1.8 s (*M* = 1.31°, *SD* = 0.55°). A positive value indicates that the target position was mislocalized in the direction of the target internal motion, irrespective of whether the internal motion was leftward or rightward in any given trial (see Data analysis for details).

A one-way repeated measures analysis of variance (ANOVA) on the saccade data showed a main effect of gap length on the size of the “saccadic illusion” (*F*(3,42) = 12.15, *p* < .001, *η* = .46). This implies that the position representation for saccades is not static but dynamically changed by internal visual motion with memory retention time. Further post-hoc pairwise comparisons indicated that the size of illusion was smaller for the 0-s gap length compared with that in all other conditions (all *p* ≤ .01 with Holm–Bonferroni correction, *d*_*z*_ ≥ .94). No significant difference was observed between all other pairs (all *p* ≥ .40). Thus, these results of the saccade tasks indicate that the effect of internal motion of a moving object (or illusion) increases as the time between the target offset and saccade initiation increases.

To compare the illusion effect on perception and saccades, we also performed planned pairwise comparisons between the size of the perceptual illusion and the saccadic illusion of each gap condition. The saccadic illusion was significantly smaller than the perceptual illusion in the 0-ms-gap condition (Dunnett's two-tailed test: *p* = .03, *d*_*z*_ = .60) but not in the other gap conditions (all *p* ≥ .57, *d*_*z*_ ≤ .23). These results indicate that when saccades were required soon after the offset of target information (< 0.6 s time gap), the saccade was less biased by internal motion than perception, which is consistent with previous observations indicating perception-action dissociation of double-drift stimuli [25]. More importantly, however, our results show that this dissociation does not appear when the delay after the target offset gets longer (> 0.6 s). In other words, a comparable saccadic and perceptual illusion size with longer time gap suggests that the accumulation of MIPS arises only after a certain period from visual inputs, which causes a large distortion of a target position for eye movements as well as for perception.

**Fig 4:**
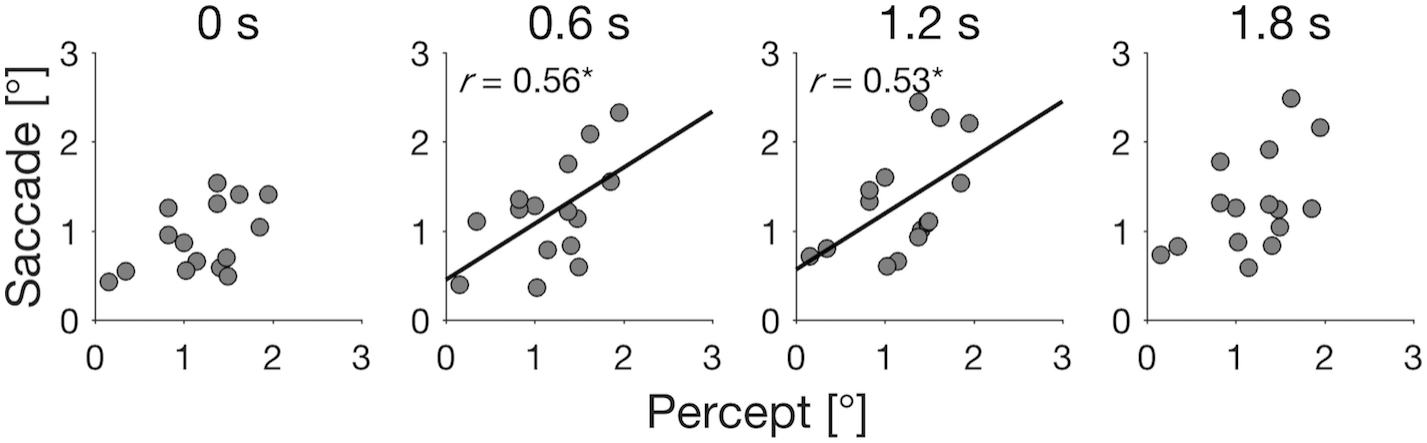
Relationship of illusion size between perception (mean 0.6-s delay) and four saccade conditions (0-s, 0.6-s, 1.2-s, and 1.8-s delay) among participants. Correlation coefficients and linear regression lines are shown in 0.6-s and 1.2-s conditions (*p* < .05).

Finally, to test the relationship between the perceived target position and the saccade landing position, we analyzed the correlation between the illusion sizes of perception and saccades in each temporal gap condition (Fig 4). The analyses showed that there was a positive correlation between the perceptual illusion (mean 0.6-s delay) and saccadic illusion in 0.6-s delay (*r* = .56, *p* = .03) and 1.2-s delay (*r* = .53, *p* = .04) conditions. The facts that the highest correlation was observed between the saccadic and perceptual illusions in the similar temporal delays suggest the possibility that the saccade and perception systems access the same target representation. If so, it is expected that similar temporal changes will be observed in the perceptual illusion. We explored this possibility in the second experiment.

## Experiment 2

In Experiment 2, we examined the temporal dependence of the perceptual illusion size while lengthening the time elapsed from the target offset as examined in the saccade task (Experiment 1). In particular, if the size of the perceptual illusion remains constant regardless of the time delay or shows different temporal development, perception and eye movement likely relies on two distinctive target position representations. On the other hand, a similar temporal change in the perceptual illusion to that seen in the saccadic illusion would support the notion that the perceptual and saccadic systems rely on a common or similar neural representation to localize moving objects.

### Methods

Sixteen paid volunteers (12 women and 4 men, aged 22–44 years: *M* = 36.7, *SD* = 6.80), 14 of whom had participated in Experiment 1 on a different day, took part in Experiment 2. All had normal or corrected-to-normal vision.

The stimuli and procedures were identical to those used for the perception task in Experiment 1, except that the vertical reference bar was presented with a randomly selected time interval of 0.3, 0.6, or 1.5 s after the target disappearance. Note that, considering the influence of visible persistence of the target after its offset, which is known to last roughly 120 ms under normal viewing conditions [33] or 60 ms for moving stimuli [34], the shortest delay in Experiment 2 was set to 300 ms; if the afterimage remains until the reference bar presentation, the target stimulus and the reference stimulus are perceived as a single image, which results in measuring the relative position of these stimuli in the same retinal reference frame (i.e., shape perception). In addition, since the illusion effect on saccades was saturated by the 0.6-s delay in Experiment 1, the two long delay conditions (1.2 and 1.8 s) of the saccade task were replaced with the 1.5-s-delay of the midpoint condition. Consequently, each participant performed 1260 trials, divided into 12 blocks, in which every four blocks consisted of all possible combinations of the three time delays, three internal motion conditions, five external motion paths, and seven reference bar locations. The data of one participant who showed no illusion effect (the same criterion as in Experiment 1) were excluded from further analyses.

### Results and discussion

**Fig 5:**
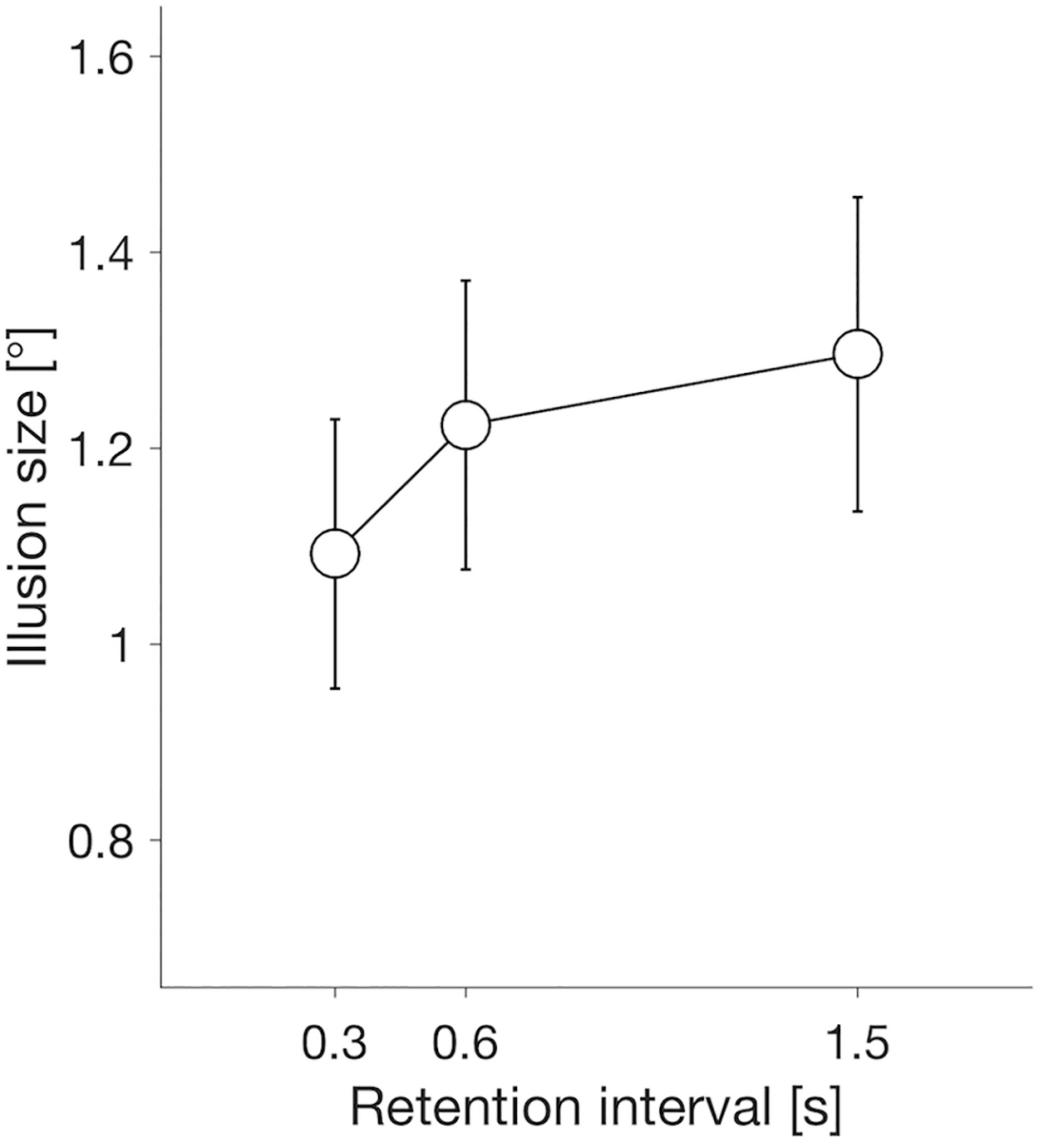
Mean illusion sizes of perception with three delay conditions (0.3, 0.6, and 1.5 s). Error bars indicate standard errors of the means (*N* = 15).

The mean sizes of the illusion with each gap length (0.3 s: *M* = 1.09, *SD* = 0.53; 0.6 s: *M* = 1.22, *SD* = 0.57; 1.5 s: *M* = 1.30, *SD* = 0.62) are shown in Fig 5. A one-way repeated measures ANOVA on the data showed a main effect of gap length on the perceived illusion size (*F*(2,28) = 4.55, *p* = .02, $\eta_{\text{p}}^{2}=.25$). A significant effect of the gap length conditions indicates that the perceived position of a motion-containing object is not static but is dynamically changed with memory retention time even when the target stimulus is no longer present. Further post-hoc pairwise comparisons showed that the illusion size with a gap length of 0.3 s was smaller than that in the other gap length conditions (all *p* ≤ .05 with Holm–Bonferroni correction, *d*_*z*_ ≥ .64). Therefore, the results of these perceptual tasks indicate that, as with the saccadic eye movements, the magnitude of the perceptual illusion is relatively small for a short period of time after the target disappearance or when its memory retention time is relatively short (≤ 0.3 s).

## General discussion

To assess the independence of the position representation of a motion-containing moving object for eye movements and perception, we examined and compared the time course of the effect of the curveball (or double-drift) illusion on saccadic eye movement and perception by controlling the memory retention intervals between the target offset and saccades (Experiment 1) and between the target offset and perceptual judgments (Experiment 2). The results showed that both the saccades and perception change in a similar time-dependent manner. In particular, the illusion effect was relatively small when saccades or perceptual judgments were made within a short period of time (0–300 ms) after the target offset, and it became larger when they were made after 600 ms.

Like the results of previous studies on MIPS, the results of the present study indicate that the position representation of a double-drift stimulus changes in a time-dependent manner, even within the same modality (perception or action). However, this temporal dependency was somewhat different from that in other types of illusions. For example, the effect of the Muller-Lyer illusion (on the length of a shaft caused by the direction of the arrow at either end) depends on the length of the presentation time, not on memory retention time, in both perception and saccades ([16,35]; see [15] for a review). The illusion effect was larger with a short presentation time and became smaller when the presentation time exceeded 200 ms. The authors considered this reduction of the illusory effect to be due to the recurrent projection of grouped information (the gist of the scene as well as the illusory context) from high-level area, which modulates the activity of the lower regions based on the actual grouping if the scene is still available, and this feedforward-recurrent loop takes about 200 ms for its first completion [16]. The same explanation could also work in the case of a single-drift stimulus (or MIPS), where the size of the mislocalization does not increase any further once it reaches its maximum size when the stimulus input is still present [24,28]. Thus, the feedforward-recurrent loop hypothesis might be compatible with the illusion effects observed in those static and single-drift stimuli. However, this hypothesis cannot be extended to the present results since the effect of double-drift stimuli increased when they were no longer in sight. In other words, if the presentation time explains the magnitude of the illusion, its effect should not increase after the target offset.

Interestingly, the temporal dependence of the double-drift stimuli observed here was not even consistent with the case observed in single-drift stimuli. It has been reported that the illusion effect of single-drift stimuli is greatest in the saccades before or just after the target disappearance and decreases as memory retention time (i.e., saccade latency) increases [24]. In contrast, we found that the illusion effect of double-drift stimuli on perception and saccades were relatively small in the response immediately after the target offset and became greater as the response delay time increased. The MIPS with a single-drift stimulus is thought to be caused by the (mis)attribution of the motion signal to the position signal when its position signal is unreliable due to the coarse information processing of peripheral vision [28] and mediated via a rapid process that completes in less than 80 ms [24]. Since a certain size of saccadic mislocalization toward the internal motion direction has been observed [25], the component of MIPS per se (i.e., the effect of internal motion alone) may also exist with double-drift stimuli. However, since the effect of single-drift stimuli does not seem to increase (at least immediately) after the target offset [24], it is unlikely that the effect of MIPS per se appeared after the delay periods in the present study.

In contrast to the static contextual illusion and MIPS, the illusion effect of double-drift stimuli increased after the visual input disappeared. One possible interpretation for this is that the position representation might be constructed by the weighted integration of an instant position representation and the history of previous position representations (i.e., trajectory). If the time before making eye movements or perceptual judgments (and hence also the time for visual information processing) is restricted, a tentative position representation, which is sufficient for localization of a target at a certain moment, is used. On the other hand, if the time is unrestricted (or if an extra memory retention time is required), a longer-lived position representation (such as a continuous trajectory path or motion direction) that reflects one's veridical perception is used, regardless of whether it is physically correct or not. Based on this hypothesis, it is reasonable that the static contextual illusion and MIPS are less susceptible to the memory retention time [24,35] because their instantaneous and long-term position representations may not be very different. Therefore, the present results can be interpreted as follows: within a short period of time after the target offset, only a local shift in the response to the internal motion affects the target representation, and, subsequently, after a sufficient period of time, the accumulated position shift due to the illusory perceived trajectory appears. In other words, the present findings imply that target representation could dynamically change by integrating with the internal representation of position history.

Another main finding of the present study is that the accumulation of MIPS (i.e., illusory position displacement of a double-drift stimulus) occurs in saccades as well as perception. In contrast, Lisi and Cavanagh [25] have demonstrated the dissociation of position representation of a double-drift stimulus between saccadic eye movements and perception; i.e., saccades were resistant to the illusion, while perception was susceptible to it. As mentioned earlier, these different results for the same stimulus may be due to the difference in the task setups between the saccade response and perceptual judgment (see also [36]). Specifically, in their study, saccades and perceptual responses were made at different times in the course of the visual process: saccades were made "as soon as possible" after a “go” signal (fixation offset), while perceptual judgments were made without a temporal constraint (or without consideration of a temporal effect). In such cases, it is plausible that saccades were made based on the position information distorted only by the internal movement at that moment, while perceptual responses were made based on integrated position information, including the previous history. Furthermore, this tendency might be emphasized by the inconsistent measurements in the previous study where the saccadic illusion was measured from the saccade landing positions and the perceptual illusion was measured from the perceived direction of the external motion; the integration of the current position with the internal representation of position history is not necessarily required in the former case, whereas the integration is indispensable in the latter case. Therefore, although Lisi and Cavanagh [25] concluded that the existence of an extra integration process with previous sensory history causes the visual processing of perception and action to diverge, the results of the present study indicate another possibility, namely that the existence of the extra integration process depends on the time allowed before making a perceptual decision or an action, rather than on the modality. Taken together, these results imply that saccadic responses and perceptual judgments are made based on a common or similar target position representation that dynamically changes over the course of information integration.

In conclusion, the present study has demonstrated a dynamic change in the position representation (illusion effect) of a double-drift stimulus (previously known to cause only perceptual illusion) for saccadic eye movements and perception. For both eye movements and perception, the illusion effect depended on the time allowed before saccadic eye movements or perceptual judgments were made. In particular, the illusion effect was smaller when saccadic and perceptual localization was done shortly after the stimulus disappearance and was larger with its memory retention time. These results can be interpreted as evidence that the accumulation of MIPS is completed only after a certain period of time and that both saccades and perceptual judgments rely on a common or similar position representation. In other words, the results of the present study suggest the importance of discussing the mechanism of the visual process for perception and action in the same temporal frame of reference.

## Supporting information

S1 Movie An example of the double-drift stimulus used in experiments. The Gabor target stimulus descends vertically with the rightward drift of the internal grating (in this example). When the stimulus is viewed in the peripheral vision (e.g., while fixating the black dot on the left), the direction of the perceived external motion deviates from its actual direction to the direction of the internal motion (which is known as the curveball illusion).
(MP4)
